# The selection and validation of reference genes for quantitative real-time PCR studies in near-isogenic susceptible and resistant tomato lines, infected with the geminivirus tomato curly stunt virus

**DOI:** 10.1371/journal.pone.0284456

**Published:** 2023-07-27

**Authors:** Mamokete Bokhale, Imanu Mwaba, Farhahna Allie

**Affiliations:** Department of Biochemistry, University of Johannesburg, Auckland Park, Johannesburg, South Africa; Deen Dayal Upadhyaya Gorakhpur University, INDIA

## Abstract

Quantitative real-time PCR (qPCR) is a sensitive and commonly used technique for gene expression profiling and provides insight into biological systems. Successful qPCR requires the use of appropriate reference genes for the normalization of data. In the present study, we aimed to identify and assess the best-suited reference genes in near-isogenic resistant (R) and susceptible (S) tomato lines infected with begomovirus Tomato curly stunt virus (ToCSV). Ten candidate reference genes namely, Actin7 (*ACT*), β-6 Tubulin (*TUB*), Ubiquitin 3 (*UBI*), Clathrin adaptor complexes medium subunit (*CAC*), Phytoene desaturase (*PDS*), Expressed protein (*EXP*), Glyceraldehyde-3-phosphate dehydrogenase (*GAPDH*), Adenine phosphoribosyl transferase-like protein (*APT1*), TAP42-interacting protein (*TIP41*) and Elongation factor 1-alpha (*EF1α*) were selected and evaluated for their expression stability in resistant and susceptible tomato leaves using the analytical tools geNorm, NormFinder, BestKeeper, and RefFinder. After ranking the reference genes from most to least stable, the results suggested that a combination of *ACT*, *EXP*, and *EF1α* in the S lines and a combination of *TIP41*, *APT1*, and *ACT* in the R line is appropriate for qPCR normalization. Furthermore, to validate the identified reference genes, iron superoxide dismutase (*SOD*), heat shock protein 70 (*HSP70*) and Glutathione-S-transferase (*GST*) were selected as targets for normalization. The relative expression of the target genes varied when normalized against the most stable reference genes in comparison to the least stable genes. These results highlight the importance of careful selection of reference genes for accurate normalization in qPCR studies.

## Introduction

South Africa is the top tomato (*Solanum lycopersicum*) producer in Southern Africa, with a yearly production of about 600.000 tonnes [1]. Most of the tomatoes produced in South Africa are consumed locally with about 3% exported to neighbouring countries [2]. To date, a number of major constraints to tomato production have been reported to be caused by viruses belonging to the Tobamovirus, Tymovirus, Tospovirus, Crinivirus and Begomovirus genera. Of particular concern is the incidence of whitefly-transmitted begomoviruses (Family: *Geminiviridae*) which has drastically increased over the recent years with reported yield losses of up to 100% [3–6].

In 1997, a devastating disease affecting tomato plants was reported in South Africa with some farmers reporting complete yield losses. Tomato curly stunt virus (ToCSV), a monopartite begomovirus was identified as the causative virus. Symptoms of ToCSV infection on tomato plants include stunting, curling and yellowing of leaves [7–10]. ToCSV bears a 76.2% nucleotide sequence similarity with Tomato yellow leaf curl-Israel (TYLCV-Is), a distinct monopartite begomovirus, reported to infect tomato plants in various tomato growing regions, with no incidence yet reported in South Africa. Since initial reports, isolates of ToCSV have been identified in neighbouring Mozambique and Zimbabwe [11, 12].

Breeding programs have developed tomato lines resistant to Tomato yellow leaf curl (TYLCV) by introgressing resistant loci from wild tomato plants into domesticated tomato plants. Some TYLCV resistant lines have been shown to carry resistance against ToCSV and these lines could be useful for the control of ToCSV in Southern Africa [10]. The allelic TYLCV resistance locus *Ty 1* and *3* originate from *S*. *chilense* accessions LA1969 and LA2779 respectively and encode for an RNA dependent RNA polymerase (RDR) [13]. Expression of RDR in *Ty-1/Ty-3* lines is believed to provide resistance through viral DNA methylation [14]. The availability of resistant and susceptible tomato plants makes tomato an ideal tool for geminivirus-host interaction studies. In order to start dissecting and understanding the mechanisms underlying geminivirus diseases, the differential expression in susceptible lines can be compared to those observed in resistant lines [15–18].

Gene expression studies using quantitative real-time polymerase chain reaction (qPCR) is the preferred method for small scale gene expression studies, as opposed to more expensive large scale and high-throughput approaches such as RNA sequencing. The reliability of gene expression studies from RT-qPCR is anchored in the selection of appropriate reference genes. These reference genes serve as internal controls that are stably expressed under the experimental conditions being investigated [19, 20]. The use of inadequate reference genes may result in incorrect expression data which would lead to the erroneous interpretation of results [21, 22]. Traditionally, “housekeeping” or “reference genes” have been selected as reference genes for normalization as their expression levels are expected to remain constant between the cells of different tissues and under different experimental conditions. Different studies have reported the selection and validation of reference genes in tomato plants during begomovirus infection [23, 24] but to date, there are no reports on the identification of suitable reference genes in tomato, in response to ToCSV infection. In this study the expression of several genes was assessed in near isogenic lines, susceptible line NIL395 and resistant line NIL396, bred for TYLCV resistance, to identify stably expressed genes to be used for qPCR studies in the tomato-ToCSV pathosystem.

## Materials and methods

### Plant growth and agroinoculation of susceptible and resistant tomato lines

Tomato curly stunt virus (ToCSV) susceptible line NIL395 (S) and resistant line NIL396 (R) tomato seeds were kindly obtained from Sakata Vegenetics R.S.A (Pty) Ltd. These R and S tomato lines were grown from seed in a controlled growth chambers at 28°C with a 16-hour light and 8-hour dark regime, until seedlings were 24 days old. Overnight cultures of *Agrobacterium tumefaciens* harbouring infectious clones of severe ToCSV (Accession: OK813888.1) were used to inoculate the 24-day old tomato seedlings (R and S) along the stem. Tomato plants were mock inoculated using *Agrobacterium* C58C1 possessing an empty pCambia2300 plasmid. Four tomato plants were agroinoculated for each line, and the experiment was independently repeated three times. At 35 days post infection, leaves were harvested and snapped frozen in liquid nitrogen. The harvested leaves were stored at -80°C until needed.

### Confirmation of ToCSV infection and viral load quantification

The symptom severity was evaluated in S and R plants over a 35-day period (S1 Fig), after which newly formed leaves just below apex were harvested, snap frozen and used in downstream expression experiments where Quantitative real-time PCR was carried out on symptomatic and positively infected samples to quantify viral load using the QuantiNova™ SYBR® Green PCR kit (QIAGEN, Netherlands) and the CFX connect™ Real-Time System (Biorad, USA). The reaction mixture was prepared in a 10 uL final volume, containing 5 μl of QuantiNova™ SYBR® Green PCR master mix, 0.5 μL of C2 primers (0.5μΜ) for ToCSV viral quantification at a final concentration of 0.5μM, and 10ng of total DNA. Real-time qPCR was run using the following cycling conditions: initial denaturation and enzyme activation at 95°C for 2 min, 40 cycles of denaturation at 95°C for 5 s, annealing/elongation at 60°C for 10 s. Similarly, a separate reaction was set up to quantify 18S to be used as a reference gene for relative virus quantification. For each reaction, melting curve analysis was performed from 65°C to 95°C at a rate of 0.05°C/s. For qPCR analysis, four infected plants and four mock-inoculated were employed along with a non-template control as well as an uninfected NIL395 and NIL396 tomato plant cDNA control (mock-inoculated sample) were included. The viral titer of ToCSV was determined using relative quantification in both S and R plants using 18S as an internal control using the equation: 2^-ΔΔCt^ method (S2 Fig).

### Total RNA extraction and cDNA synthesis

Total RNA was extracted from infected and mock inoculated leaf tissue using quick RNA miniprep kit (Zymo Research, USA) as per manufacturers instruction. The extracted RNA was eluted in 50μl of RNase-free water and quantified using Nanodrop^TM^ spectrophotometer (ThermoFisher Scientific, USA) and for integrity check, RNA was run on a 1.5% agarose electrophoresis gel prepared in 0.5x TBE buffer. Total RNA was converted to cDNA using random hexamers and oligo (dT)18 primers contained in Maxima H Minus First Strand cDNA Synthesis kit RT-qPCR (Thermo scientific, USA) as recommended by the manufacturer. The synthesized cDNA was diluted 1 in 10 with nuclease free PCR water before qPCR analysis.

### Primer design

Nucleotide sequences of the candidate genes actin7 (*ACT*), Β-6Tubulin (*TUB*), ubiquitin 3 (*UBI*), clathrin adaptor complexes medium subunit(*CAC*), phytoene desaturase (*PDS*), expressed protein (*EXP*), glyceraldehyde-3-phosphate dehydrogenase (*GAPDH*), Adenine phosphoribosyl transferase-like protein (*APT1*), TAP42-interacting protein (*TIP41*) and Elongation factor 1-alpha (*EF1α*) and targets genes iron superoxide dismutase (*SOD*), heat shock protein 70 (*HSP70*) and glutathione-S-transferase (*GST*) used in this study were obtained from the Sol Genomics Network (https://solgenomics.net) database. Primers were designed of each gene **(Table 1)** using Integrated DNA technologies Primer Quest Tool (http://www.idtdna.com/pages/scitools) and synthesized by Inqaba Biotechnical industries (Pretoria, South Africa). Primer efficiencies were obtained for each candidate and target gene using dilutions of cDNA and a standard curve was generated from five 10-fold dilution series on The CFX connect™ Real-Time System (Bio-Rad, USA). The amplification efficiency of the primers was calculated using the equation: PCR efficiency = (10)^-1/slope^– 1 according to the Minimum Information for Publication of Quantitative Real-Time PCR Experiments (MIQE) guidelines for qPCR experiments [20].

**Table 1:** Primer sequences for reference and target genes used for qPCR in this study.

Gene name	Primer sequence	Amplicon size (bp)	Accession number ^a^
Actin 7 (*ACT*)	GGTATCCACGAGACTACCTACA TGCTCATACGGTCAGCAATAC	127	Solyc11g005330.2
Β-6Tubulin (*TUB*)	GCTACCTGTGGAAGGTTTGT GGACGGAAGATCTGTCCATAAG	101	Solyc10g086760.2
Ubiquitin 3 (*UBI*)	CTTCGTAAGGAGTGCCCTAATG GCCTCCAGCCTTGTTGTAA	117	Solyc01g056940.3
Clathrin adaptor complexes medium subunit (*CAC*)	CCTCCGTTGTGATGTAACTGG ATTGGTGGAAAGTAACATCATCG	173	SGN-U314153 (Expósito-Rodríguez et al., 2008)
Phytoene desaturase (*PDS*)	CAAGACCAGAGCTGGACAATAC CAAACCTGCACCAGCAATAAC	119	Solyc03g123760.3
Expressed protein (*EXP*)	GCTAAGAACGTGGACCTAATG TGGGTGTGCCTTTCTGAATG	183	SGN-U346908 (Expósito-Rodríguez Exposito-Rodriguez et al., 2008)
Glyceraldehyde-3-phosphate dehydrogenase *(GAPDH)*	GGGTTGCTCTCCAAAGAAATG CTGGCCGTGTACACTATCATAC	107	Solyc03g111010.3
Adenine phosphoribosyl transferase-like protein (*APT1*)	TCAGTGTGGTTGCAGGTATTG CCCAGGTAACTTCTTGGGTTTC	110	Solyc04g077970.3
TAP42-interacting protein (*TIP41*)	ATGGAGTTTTTGAGTCTTCTGC GCTGCGTTTCTGGCTTAGG	235	SGN-U584254 (Expósito-Rodríguez et al., 2008)
Elongation factor 1-alpha (*EF1α*)	GGCCAGATTGGAAACGGATACTTACCTGAACGCCTGTCAA	105	Solyc06g005060.3
Iron Superoxide dismutase (*SOD*)	GGCCTGGAATCATCAGTTCTT GCTGCAGCTGCCTTAAATTC	138	Solyc06g048410.3
Glutathione-S-transferase (*GSH*)	TGGGTTCTACTGCTGGTTTC TTAGCCACACTGTCCCTTTG	117	Solyc07g056420.4
Heat shock protein (*HSC 70*)	CACCACTTTCTCTTGGGTTAGA CCGGGTTGGTTATCAGAGTAAG	121	Solyc06g076020.3

^a^ Accession numbers from Sol genomics network (SNG or Solyc: https://solgenomics.net/)

### Quantitative Real–Time PCR

Gene expression was assessed using a qRT-PCR assay performed on a CFX connect™ Real-Time System (Bio-Rad, USA). The RT-qPCR reaction was performed in a 10 uL volume and included 2 μl of diluted cDNA, 5 μl of 2X Luna Universal qPCR SYBR Green mastermix (New-England Biolabs, USA) and primers to a final concentration of 500 nM. The following amplification conditions were used: initial denaturation and enzyme activation at 95°C for 60 s, followed by 40 cycles at 95°C for 15 s, 60°C for 30 s. Each assay included three technical replicates. The specificity of amplification was assessed by a single peak in the melting curve analysis. For melting curve analysis, a dissociation step cycle was performed at 65°C for 0.5 s followed by a gradual increase of 0.05°C for 0.5 s until 95°C. The raw data generated from this study is stored and available through the following DOI: https://doi.org/10.1016/j.dib.2020.105750.

### Evaluation and validation of expression stability of candidate reference genes

Four analytical tools were used to rank expression stability of genes namely, NormFinder [25], geNorm [26], BestKeeper [27] and RefFinder [28]. The target genes *SOD*, *HSP70* and *GST* were used to validate the reference genes ranked as the most and least stable in the entire study. Relative quantification using 2^-ΔΔCT^ [29] was used to calculate the relative expression of the target genes after ToCSV infection. Differential gene expression was analysed using the Student’s t-test. For S plants, correlation analysis was performed to compare the differential gene expression normalized against the 3 best reference genes to that normalized against pairs of reference genes. Graphical representations and statistical analysis were carried out using Microsoft Excel. The *p*-value for significance was set at below 0.05.

## Results

### Expression profiling of candidate reference genes

Ten genes *ACT*, *EF1α*, *EXP*, *CAC*, *TUB*, *PDS*, *APT1*, *TIP41*, *GAPDH*, and *UBI*, previously identified as stably expressed in tomato in response to various biotic and abiotic stresses were selected as candidate reference genes. Prior to determining their expression levels, the specificity of each primer pair was determined by a single peak in the melting curve analysis and further confirmed by a single band for each RT-qPCR amplicon on 2% agarose gel electrophoresis (S3 Fig). The PCR efficiency of each primer pair was obtained from a standard curve. All candidate reference genes had an amplification efficiency of 91.4% and higher and a correlation coefficient above 0.991 (S4 Fig).

**Fig 1:**
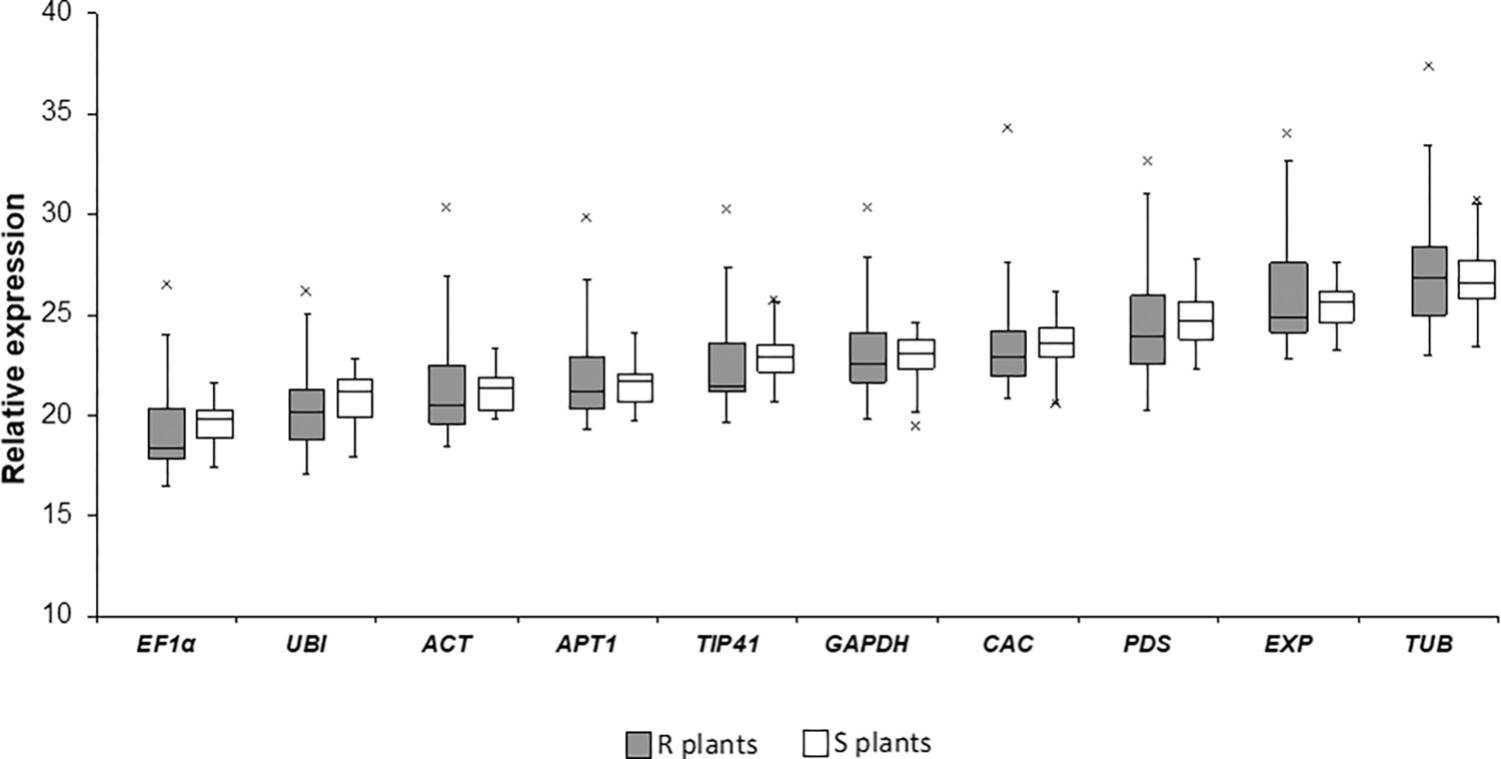
Box plot representing the Cq values of ten candidate reference genes in R and S plants in both infected and mock plants (n = 24). The box indicates 25% and 75% percentile, while the central horizontal line shows the median. The whiskers represent the maximum and minimum values, whereas the asterisks indicate the outliers.

The expression levels of the candidate genes were determined in infected R and S plants, at 35 dpi, relative to their respective expression in mock inoculated R and S tomato plants. Average cycle quantification (Cq) values were calculated to reveal the expression levels of reference genes in both tomato lines (Fig 1). The average Cq value of the candidate genes were within the recommended Cq value of 15 to 30 [31] ranging from 19.69 (*EF1α*) and 26.78 (*TUB*) in R plants and from 19.22 (*EF1α*) and 27.06 (*TUB*) in R plants **(**Fig 1**).** *EF1α* had the highest accumulation of transcript with an average Cq value below 20 in both tomato lines while reference genes *TUB* was shown to be lowly expressed with Cq values 26.78 and 27.06 in R and S lines respectively.

### Expression stability of candidate reference genes using BestKeeper

The BestKeeper algorithm was used to rank the stability of the reference genes according to the standard deviations (SD), coefficients of variance (CV) and coefficient of correlation (*r*) [27]. The ranking order of the reference genes was from the most stable to the least stable using coefficient of correlation *r* **(**Tables 2 and 3**).** For S plants, the 3 candidate reference genes best suited for normalization with *r* values closest to 1 were *EF1α*, *ACT*, *EXP* and for R plants, *ACT*, *TIP41*, *APT1* had *r* values closest to 1. Candidate reference genes that were the most variable with the lowest *r* values were *TIP41*, *GAPDH* and *UBI* for S plants and *CAC*, *GAPDH* and *UBI* for R plants.

**Table 2:** Gene stability values and rankings for R plants.

Gene Rank	BestKeeper		geNorm		NormFinder		RefFinder	
	Rank	*r*	Rank	Stability (M)	Rank	Stability (M)	Rank	Stability value
*ACT*	1	0.989	1	0.968	2	0.176	1	1.50
*APT1*	2	0.987	2	0.971	3	0.214	2	1.68
*TIP41*	3	0.982	3	1.002	1	0.162	3	3.22
*EXP*	4	0.982	4	1.007	4	0.288	5	4.76
*EF1α*	5	0.961	5	1.129	5	0.314	4	4.40
*TUB*	6	0.951	9	1.501	9	0.553	10	9.24
*PDS*	7	0.937	6	1.382	7	0.454	7	6.64
*CAC*	8	0.932	8	1.428	6	0.439	9	7.48
GAPDH	9	0.904	7	1.411	10	0.575	8	6.96
UBI	10	0.848	10	1.625	8	0.552	6	5.62

**Table 3:** Gene stability values and rankings for S plants.

Gene Rank	BestKeeper		geNorm		NormFinder		RefFinder	
	Rank	*r*	Rank	Stability (M)	Rank	Stability (M)	Rank	Stability value
*ACT*	1	0.972	1	0.717	1	0.082	1	1.00
*EF1α*	2	0.968	3	0.732	2	0.119	2	2.21
*EXP*	3	0.960	2	0.727	4	0.157	3	2.28
*CAC*	4	0.948	4	0.806	3	0.156	4	4.60
*TUB*	5	0.917	6	0.994	5	0.242	9	8.71
*PDS*	6	0.870	7	1.020	7	0.255	6	6.64
*APT1*	7	0.825	5	0.979	8	0.280	5	5.00
*TIP41*	8	0.784	8	1.053	6	0.252	6	6.09
GAPDH	9	0.743	9	1.158	9	0.285	8	7.90
UBI	10	0.706	10	1.212	10	0.327	10	9.46

### Expression stability of candidate reference genes using geNorm

**Fig 2:**
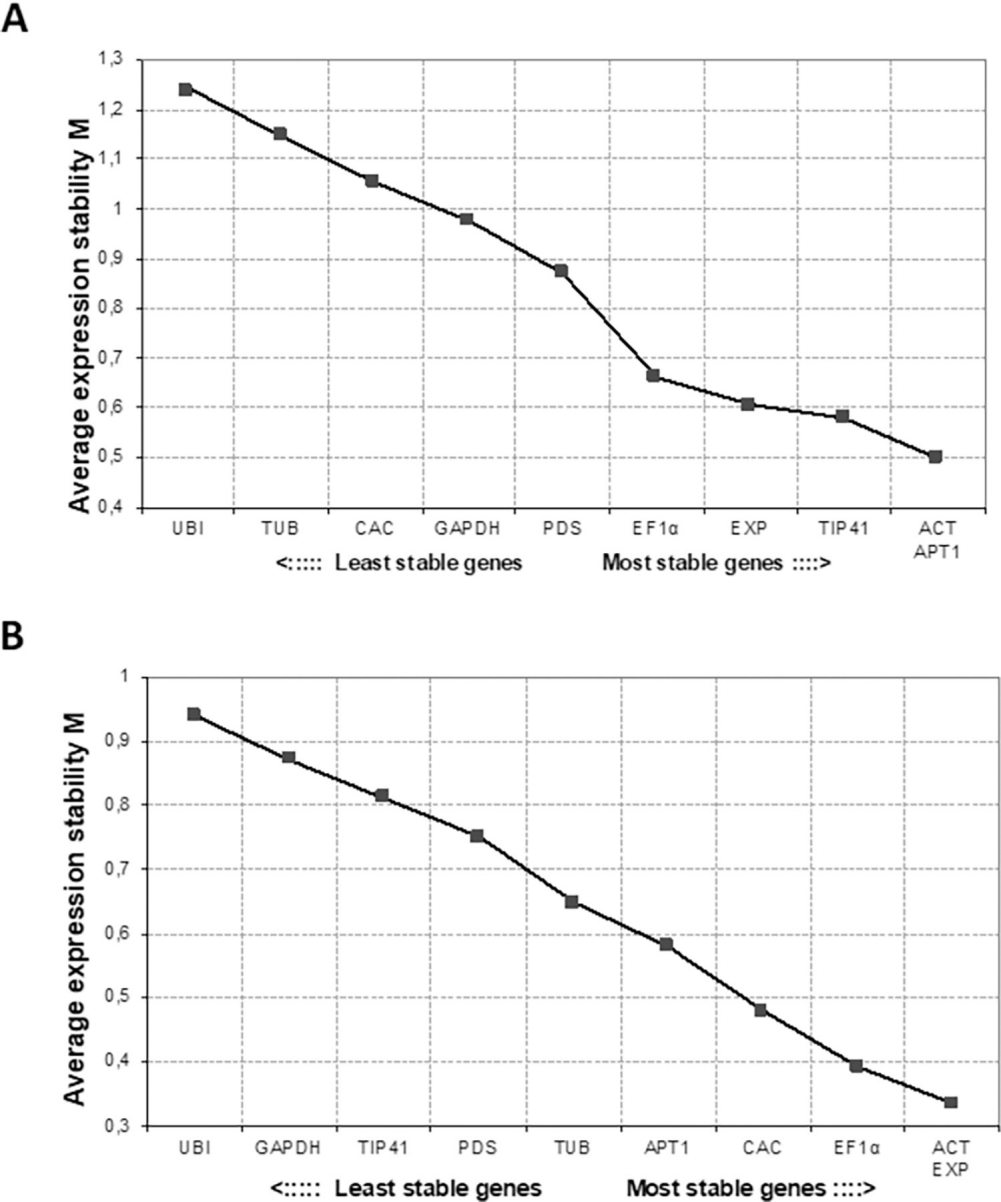
geNorm analysis of average stability (M) of reference genes. Tomato M values are indicated for both (A) R plants and (B) S plants, n = 24. The least stable reference genes are shown on the right while the most stable reference genes are on the left. M-value is inversely proportional to gene stability.

The expression stability of candidate genes was analysed using geNorm, which ranks candidate reference genes according to the measure M, from the most stable (lowest M value) to the least stable (highest M value), with M values of above 1.5 regarded as unacceptable levels of expression variability [26]. In S plants, the most stable reference genes were *ACT*, *EXP* and *EF1α* and the least stable *UBI*, *GAPDH* and *TIP41*. In R plants, *ACT*, *APT1* and *TIP41* were most stable with *UBI*, *TUB* and *CAC* being least stable reference genes **(**Fig 2**).** The successive elimination of the least stable reference genes was based on the highest M values.

**Fig 3:**
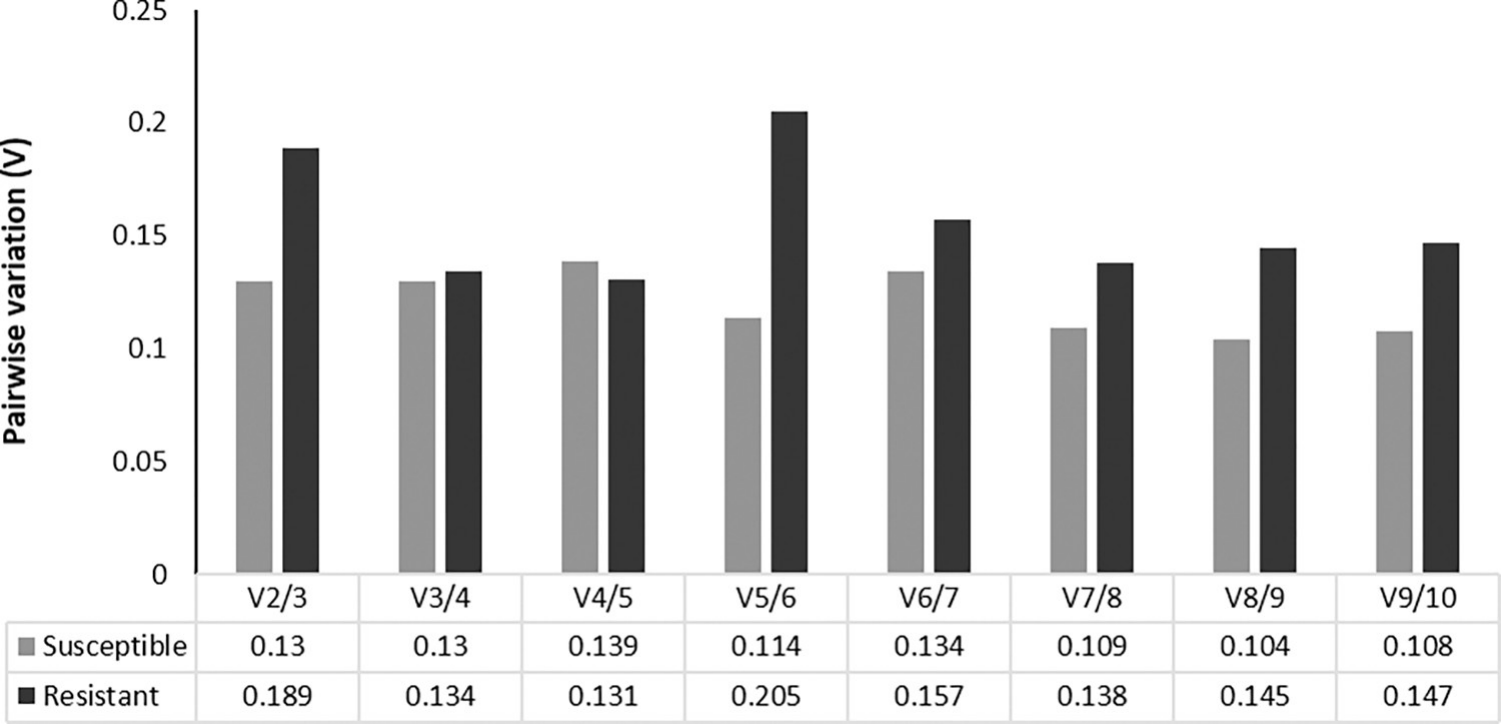
geNorm analysis of selected reference genes in the tomato-pathosystem. Analysis of Pairwise variation (Vn/Vn+1) was between the normalization factors NFn and NFn+1 to determine the optimal number of reference genes by geNorm required for accurate normalization of qPCR data. A stability maximum of 0.15 is used to determine whether more reference genes should be included for normalization.

geNorm can estimate the optimal number of reference genes to be used for normalization by calculating the pairwise variation (V) of the normalization factor (NF). Pairwise variation of 2 reference genes compared to three reference genes (V2/3) for value of R plants was more than the proposed cut-off value of 0.15 indicating that 3 most stable reference genes should be used for accurate RT-qPCR normalization in R plants. In S plants, the V2/3 values were lower than 0.15 suggesting that the two most stable reference genes, *ACT* and *EXP* are optimal for normalization **(**Fig 3**).**

### NormFinder analysis

**Fig 4:**
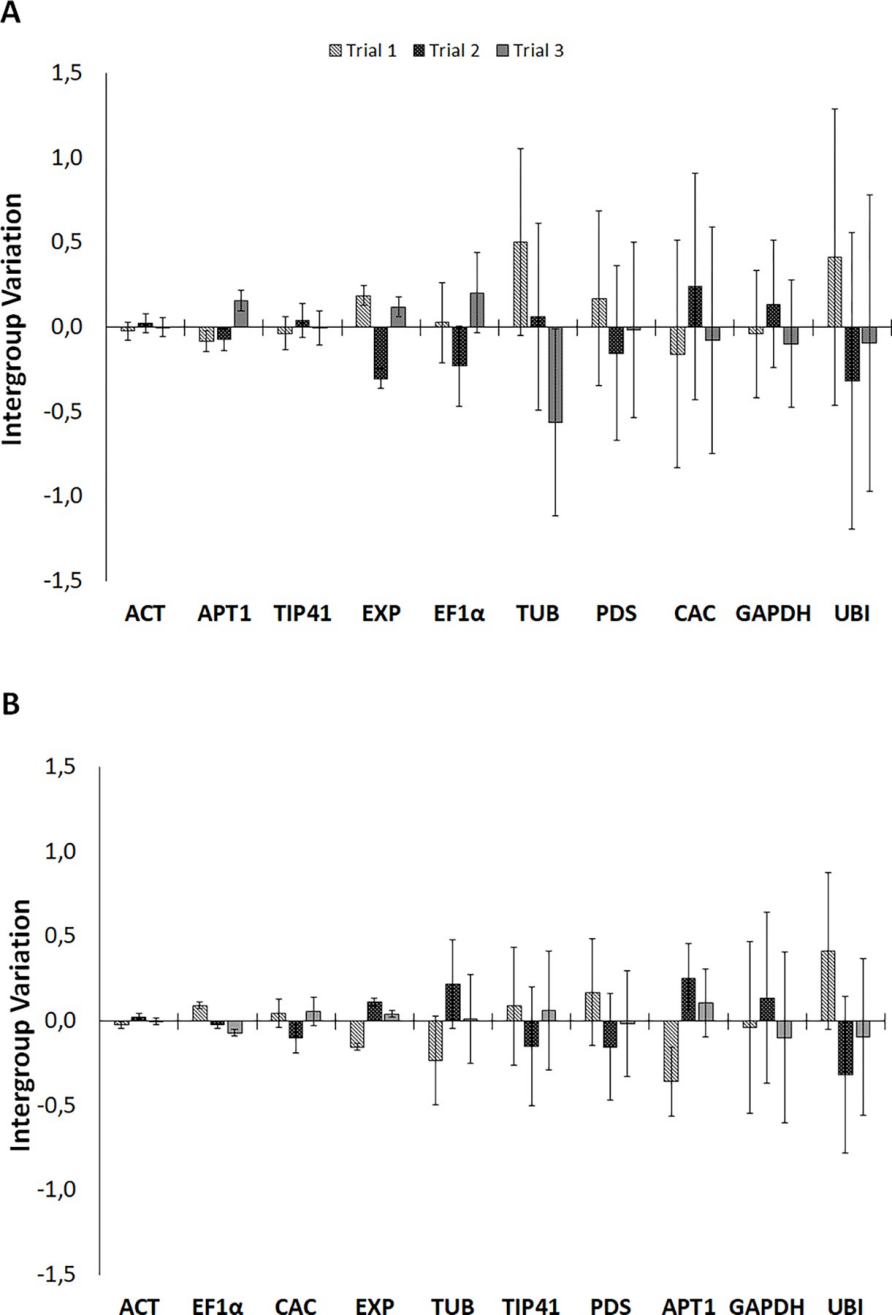
NormFinder analysis estimates intra and intergroup variations for each candidate reference gene between A and B. Intergroup variations are represented on the y-axis and reference genes on the x-axis. Error bars show the intragroup variations for A (R plants) and B (S plants), intergroup variations differences closer to 0 and minimal error depict higher expression stability.

NormFinder ranks the most suitable reference gene as the one that has the lowest stability value (SV). This analysis tool also considers variation in gene expression that occurs across experimental conditions looking at intergroup and intragroup variations across the candidate reference genes [25]. The results for S plants on NormFinder indicated that *ACT had* the lowest SV and was the most stably expressed, followed by *EF1α and APT1*, whilst *GAPDH* and *UBI* were the least stably expressed reference genes. *ACT* and *EF1α* were classified as the best combination reference genes. For R plants, *TIP41* had the lowest SV value hence was the most stable, followed by *ACT* and *APT1* whilst *UBI*, *TUB* and *GAPDH* were the least stable. *ACT and APT1* were the most suitable reference genes combination for R plants **(**Fig 4**).** Reference genes *ACT* and *TIP41* which had the lowest SV values in both R and S plants, had the lowest inter and intragroup variation and commonly to R and S plants, *GAPDH* and *UBI* had the highest inter/intragroup variation **(**Fig 4**).**

### RefFinder analysis

RefFinder was used to obtain a final ranking of candidate reference genes, from the most to the least suitable reference gene given that it integrates algorithm used by geNorm, NormFinder, BestKeeper and comparative delta CT method to rank candidate reference genes [28]. Analysis by RefFinder ranked *ACT*, *EF1α* and *EXP* as most stable reference genes for S plants and *ACT*, *APT1* and *TIP41* as most stable reference genes for R plants. The transcript abundances of *TUB*, *UBI*, *GAPDH* was unstable in all S plants, and for R plants, expression of *GAPDH*, *CAC* and *TUB* was found to be unstable. **(Tables 2 and 3**).

Taken together with the RefFinder outputs, all four algorithms used in this study identified *ACT*, *APT1* and *TIP41* as the most stably expressed reference genes in R plants (**Table 2**). In S plants, all but NormFinder found *ACT*, *EF1α* and *EXP* to be stably expressed whilst NormFinder ranked *CAC* above *EXP* (**Table 3**).

### Validation of candidate reference genes using genes

**Fig 5:**
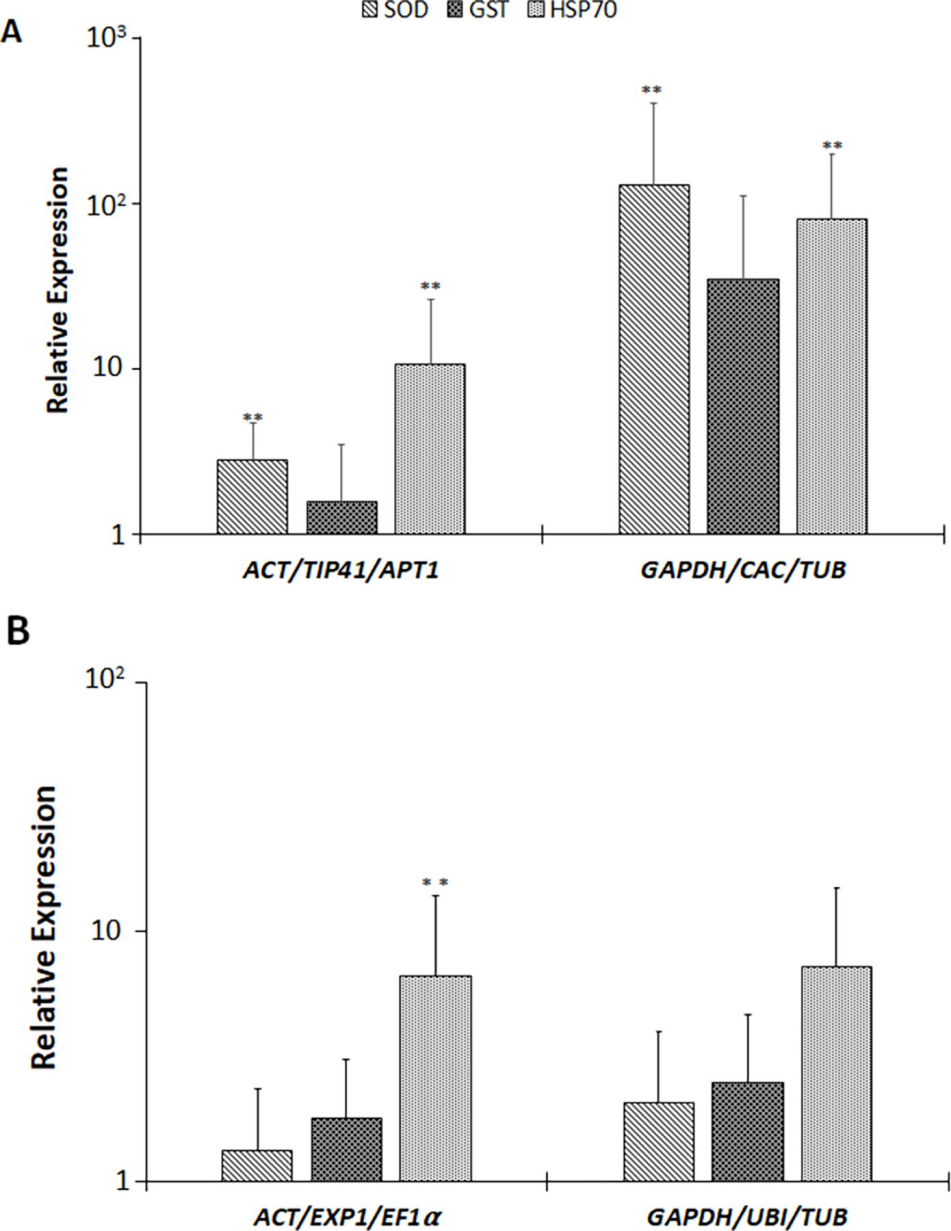
Relative expression of target genes SOD, GST and HSP70 in S and R lines infected with ToCSV. Gene expression for R plants (A) and S plants (B) was normalized against the most and least stable reference genes for normalization as indicated by the Y-axis. Means were calculated from 12 replicates; error bars represent standard deviation. Asterisks (**) indicate significant differences set at p < 0.05 determined by the Student’s t-test.

To validate the identified reference genes, the expression of iron superoxide dismutase (*SOD*), glutathione-S-Transferase (*GST*) and heat shock protein 70 (HSP) was sought using RT-qPCR and *ACT*, *EF1α* and *EXP* for S plants and *ACT*, *APT1* and *TIP41* for R plants were used for normalization. Normalization using the three most suitable reference genes revealed that the expression of *GST* was unchanged in both R and S plants, the expression of *HSP70* was upregulated by about 15-fold in R and about 6fold in S plants. The expression of *SOD* remained unchanged in S plants, whilst in R plants, *SOD* was upregulated by 3-fold **(**Fig 5**).**

Normalization using the best three reference genes was compared to normalization using the least suitable genes *GAPDH*, *CAC* and *TUB* for R plants and *TUB*, *UBI* and *GAPDH* for S plants. In R plants, there was an apparent overestimation and increase in variability of gene expression when expression of *SOD* and *HSP70* was normalised against the three least suitable genes, *SOD* and *HSP70* were upregulated 180- and 80-fold respectively. In S plants, normalization with the three least suitable genes resulted in an increase in variance measured by the increased standard deviation values and the upregulation of *HSP70* measured when normalised against 3 most suitable reference genes was masked **(**Fig 5**).**

**Fig 6:**
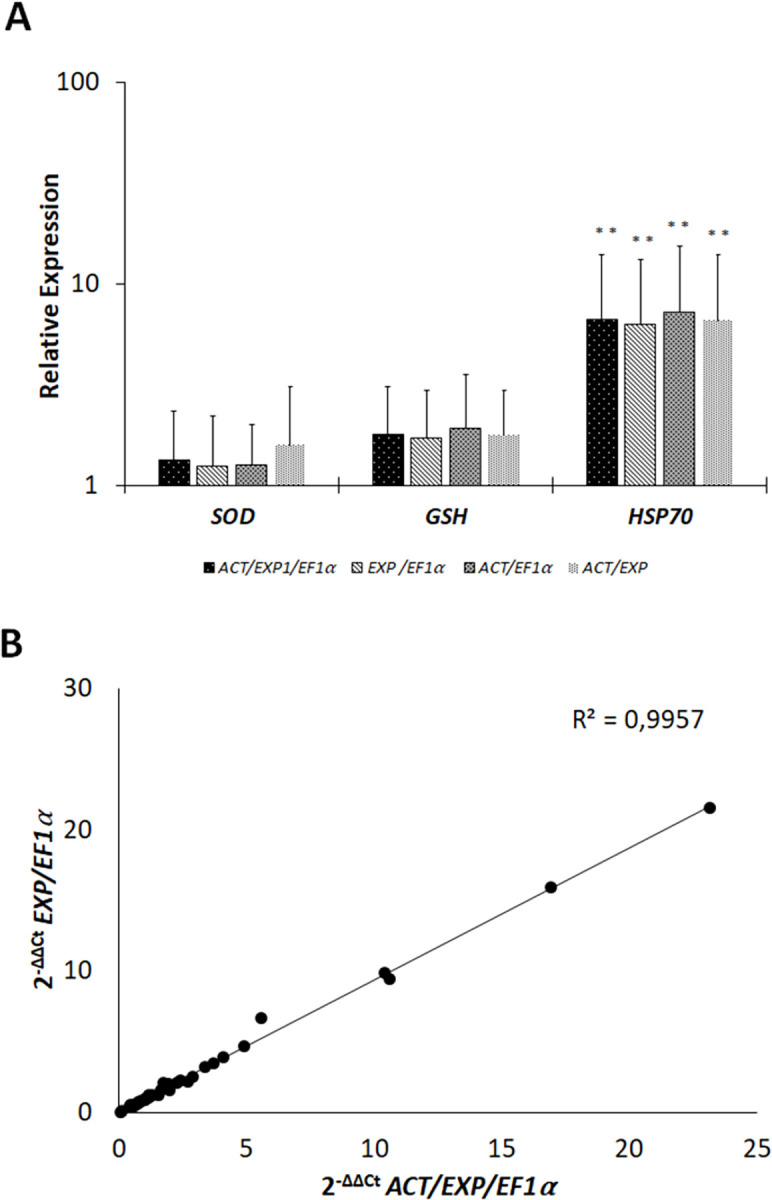
Normalization of *SOD*, *GST* and *HSP70* using different combinations of reference genes in S plants. (A) Gene expression was normalized against pairs of most stable reference genes and compared to normalization against the three most stable genes, as indicated by the Y-axis. Means were calculated from 12 replicates; error bars represent standard deviation. Asterisks (**) indicate significant differences set at p < 0.05 determined by the Student’s t-test. (B) scatter plot of correlation between gene expression normalized against EXP/EF1α to gene expression normalized against *ACT*/ *EXP*/*EF1α* (n = 24, 12 infected and 12 mocks).

geNorm indicated based on its V2/3 values that 2 reference genes were sufficient for normalization in S plants. Whilst NormFinder identified *ACT*/*EF1α* as the best combination pair, geNorm identified *ACT*/*EXP*. To confirm which pair of reference genes would be sufficient to normalize gene expression in S plants, the expression of *SOD*, *GST* and *HSP70* normalized against the three suitable genes *ACT*, *EF1α* and *EXP*, was compared to expression normalized against the reference genes in pairs. The expression of *SOD*, *GST* and *HSP70* normalized against each pair was comparable to that normalized against the best three reference genes, as the expression of *GST* and *SOD* was unchanged, and the expression of *HSP70* was upregulated by about 6-fold (Fig 6). There was a high correlation observed when normalization against each pair *ACT/EXP*, *ACT*/*EF1α* and *EF1α/EXP* was compared to normalization against the three best reference genes, confirming the suitability of using two reference genes for normalization (S5 Fig). Normalization against *EF1α/EXP* which showed the strongest correlation and the lowest variance **(**Fig 6**).**

## Discussion

Gene expression studies in plant-virus interactions provide insights into the mechanism of disease susceptibility, disease resistance and highlights key players involved in response to infection. Real-time PCR is the most widely used technique to profile gene expression patterns. The first step of real-time PCR experiments for gene expression studies requires the selection and validation of reference genes for data normalization [19, 30]. There are many reports on validation of candidate reference genes in tomato in response to various stimuli [23, 24, 31–36]. In all these studies carried out in tomato plants, fruits and seeds, the most suitable reference genes that were identified varied and no two genes were found to be stably expressed across all experimental conditions. Until a stably expressed universal reference gene under, any experimental conditions, has been identified, it is critical to evaluate the stability of several candidate reference genes for in each experimental system before its use in any downstream qPCR analysis.

In this study, the expression of ten of the most commonly used candidate reference genes was assessed in ToCSV infected near-isogenic R and S tomato plants, using BestKeeper, geNorm, NormFinder and RefFinder to select and rank the most suitable reference genes. Each of these statistical tools uses different algorithm to select the reference genes. BestKeeper determines the most suitable reference genes by calculating the correlation of coefficient (*r*) value, standard deviation, and coefficient of variance of Cq values [27]. geNorm measures stability M values and determines the optimal number of reference genes required for normalization by calculating pairwise variation (Vn/n+1). NormFinder combines stability M values and inter/intragroup variation in gene expression of the candidate genes, to select the most appropriate reference genes [25, 26]. It is not uncommon that seeking reference genes using any of these tools leads to different results, given the different algorithms used by each tool. The recommendation that more than one tool be used to determine the most suitable reference gene for a study led to the development of the internet based tool named RefFinder, which combines the ΔCt method, BestKeeper, geNorm and NormFinder algorithm into one tool [28]. In cases where no consensus on the chosen reference genes and their ranking can be reached using BestKeeper, Normfinder and geNorm, outputs from RefFinder trump that of the others. In R plants, all 4 tools identified *ACT*, *APT1* and *TIP41* as most stably expressed. Whilst BestKeeper and geNorm ranked *ACT* first and *TIP41* third, NormFinder ranked *TIP41* first and *APT1* third and RefFinder ranked *TIP41* first and *ACT*1 third. For S plants, there was a consensus on *ACT* and *EF1α* being stably expressed, with NormFinder identifying *CAC* as the third reference gene and BestKeeper, geNorm and RefFinder selected *EXP* as the third reference gene. In order to validate the selected reference genes, the expression of *SOD*, *GST* and *HSP70* which have all been previously linked to geminivirus stress response [24, 37–41] was normalized against *ACT*, *APT1* and *TIP41* in R plants and against *ACT*, *EXP*, and *EF1α* in S plants. Expression of *SOD* and *HSP70* was upregulated in ToCSV infected R plants whilst expression of *GST* remained unchanged and in S plants, expression of *HSP70* was upregulated in infected plants while that of *GST* and *SOD* remaining unchanged.

Whilst the algorithm used by RefFinder encompasses the algorithms of BestKeeper, geNorm and NormFinder, each of these tools have their own merit. The M stability values obtained from geNorm and NormFinder and the *r* values, CV and SD from BestKeeper can together be used as a pre-screening measure of the stability of expression of the candidate genes and by measuring V2/3 values, geNorm can help determine the optimal number of reference genes required for normalization. In this study, geNorm indicated that 2 reference genes were sufficient for normalization in S plants in contrast to 3 reference genes required for R plants. Whilst *ACT* was identified as the most stable reference gene in both R and S lines, normalization against a single gene is not recommended for reliable RT-qPCR normalization [19]. The use of two reference genes in S plants as suggested by geNorm was tested by normalizing the expression of *SOD*, *GST* and *HSP70* against pairs of reference genes and normalization against *EXP* and *EF1α*, had the highest correlation when compared to normalization against all 3 reference genes, suggesting that *EXP* and *EF1α* could be sufficient for normalization in S plants.

The results presented here emphasize the importance of selecting stable reference genes for accurate interpretation of qPCR for each experiment. Previous research into identifying suitable reference genes in tomato leaves in response to the bipartite begomovirus tomato chlorotic mottle virus (ToCMoV) identified *TIP41* and *EF1α* as the most stable reference genes in both resistant and susceptible tomato cultivars [23]. In response to the monopartite begomovirus TYLCV, *ACT*, *GAPDH* and *UBI* were identified as the most suitable reference genes [24]. In this research, *ACT* alone was identified as suitable to be used in both R and S plants whilst the other two suitable reference genes *UBI* and *GAPDH* were line specific and amongst the least suitable in both R and S plants highlighting that viruses belonging to the same genus do not always trigger the same response in their host. Despite the genetic background that both R and S lines share, *ACT* was the only common gene stably expressed in ToCSV infected R and S lines. These near isogenic lines only differ in the presence or absence of the resistant locus *ty1*, and the difference in stable reference genes identified in these lines further emphasizes the need to seek reference genes, specific for each experiment in order to obtain accurate results. This work is the first to identify reference genes in response to ToCSV infection in tomato. Given the spread in occurrence of ToCSV from South Africa to its neighbouring countries, the identification of these genes would provide an ideal tool for gene expression studies in response to ToCSV in the broader Southern African region.

## Conclusion

This study is the first to select and evaluate reference genes for gene expression analysis in ToCSV infected resistant and susceptible tomato plants. Four different software tools were used, and each software gave distinct outputs for each of the cultivars i.e. susceptible and resistant. Using RefFinder, provided the most stably expressed reference genes for each cultivar. *ACT*, *EXP* and *EF1α* were the most stably expressed genes in the susceptible cultivar and *TIP41*, *APT1* and *ACT* were the most stably expressed in the resistant cultivars. The identified reference genes were used to analyse the expression of *SOD*, *GST* and *HSP70* genes, and were confirmed to be suitable reference genes for the normalization of target gene expression. The results further validate the need for selecting specific reference genes for the accurate normalization of gene expression in each virus-host pathosystem.

## Supporting information

S1 Fig (PDF)

S2 Fig (PDF)

S3 Fig (PDF)

S4 Fig (PDF)

S5 Fig (PDF)
