# Supplementary figures and images for: The selection and validation of reference genes for quantitative real-time PCR studies in near-isogenic susceptible and resistant tomato lines, infected with the geminivirus tomato curly stunt virus
Source: PLoS One. 2023 Jul 27;18(7):e0284456. doi: 10.1371/journal.pone.0284456 (PMC10374155; doi:10.1371/journal.pone.0284456)

## Additional Supporting file 1

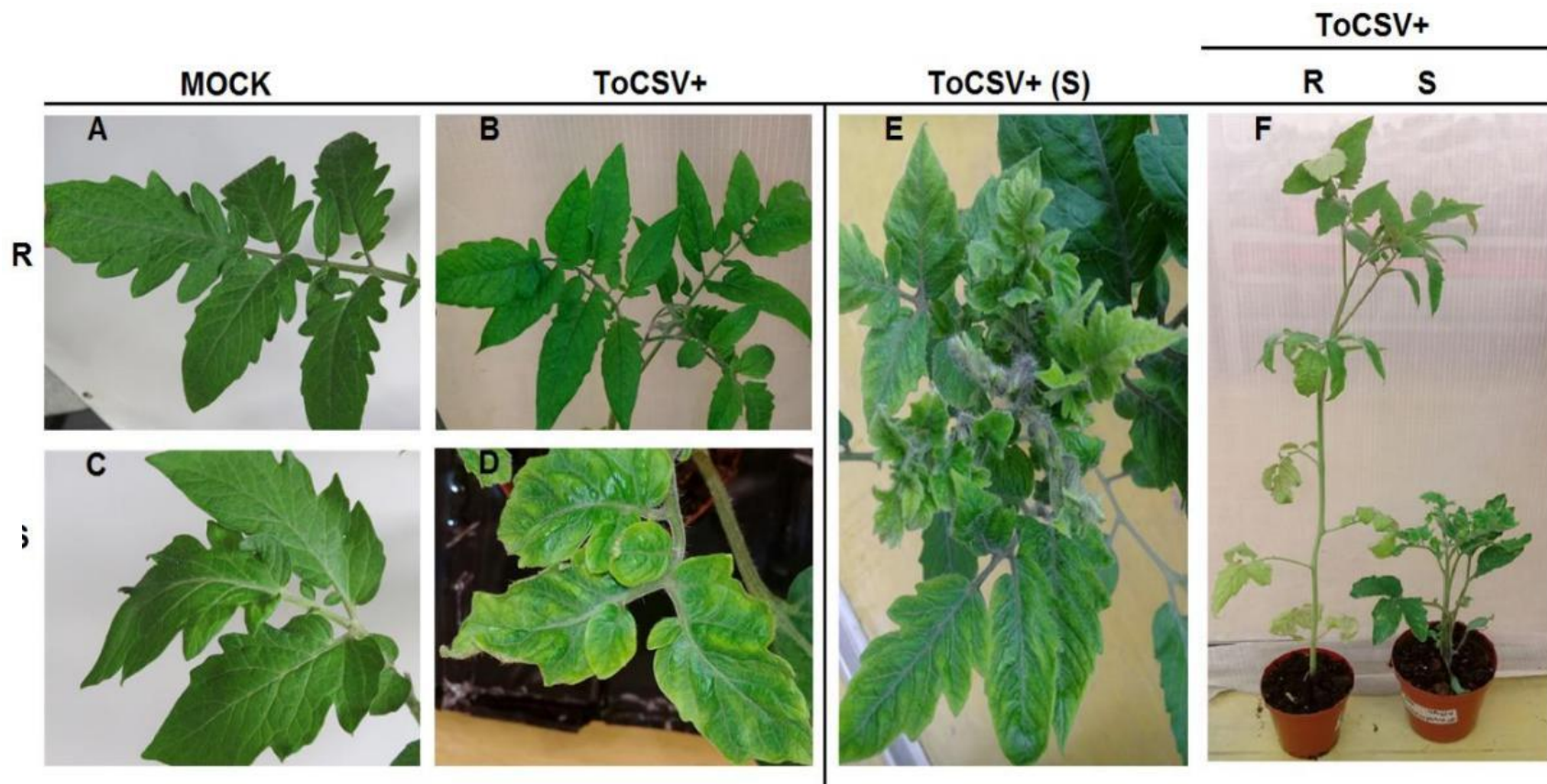

Supplement: S1 Fig — (PDF) [file pone.0284456.s001.pdf]

## Additional Supporting file 2

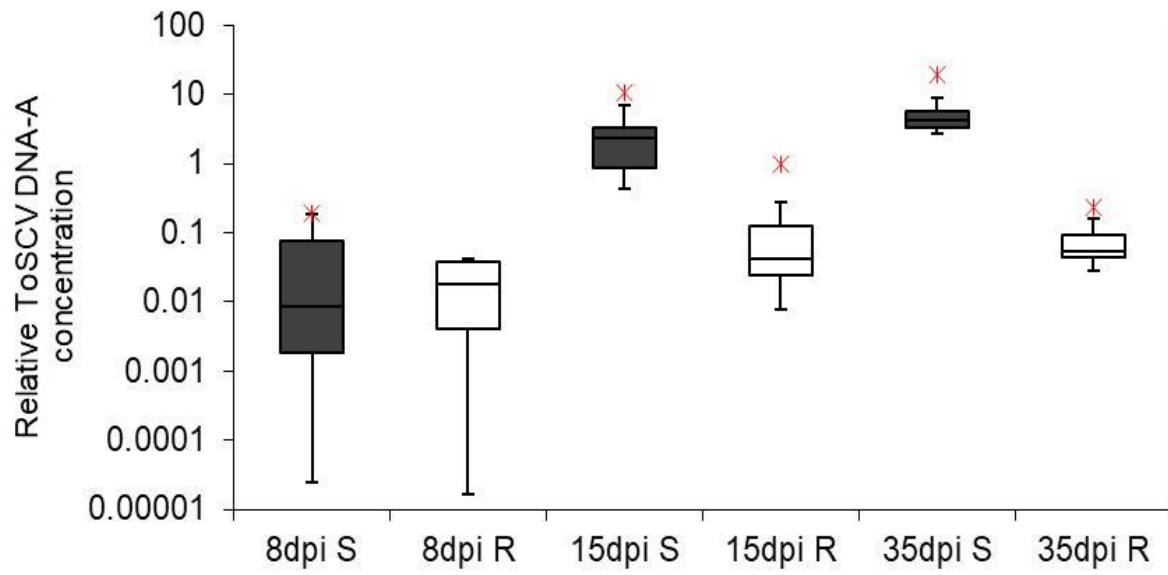

Supplement: S2 Fig — (PDF) [file pone.0284456.s002.pdf]

Additional Supporting Figure 4

**A**

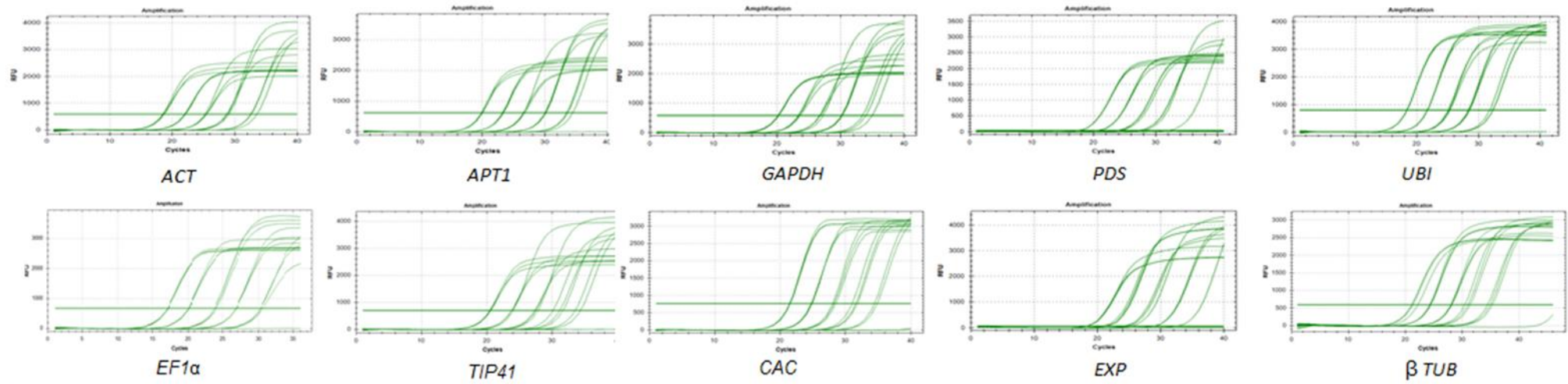

**B**

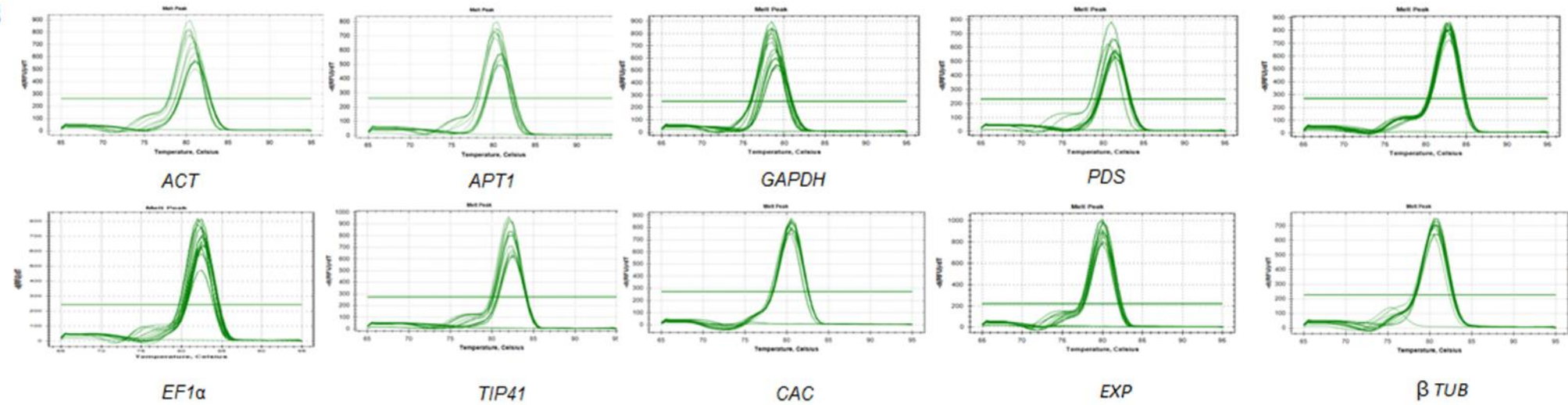

C

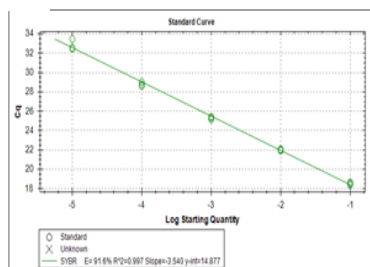*ACT*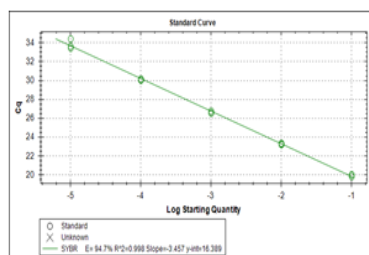*APT1*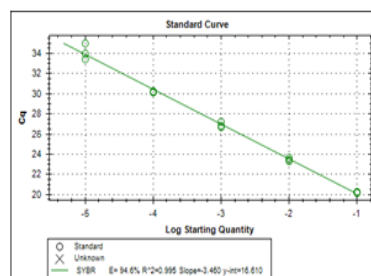*GAPDH*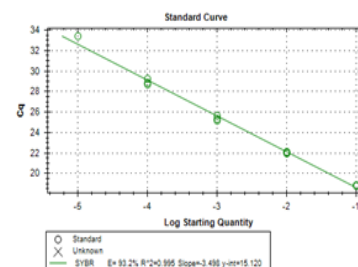*PDS*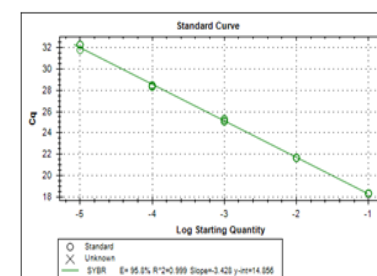*UBI*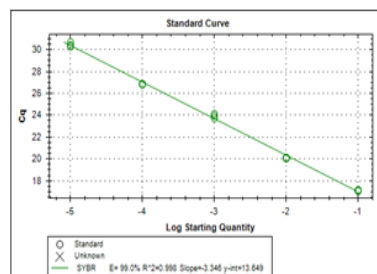*EF1α*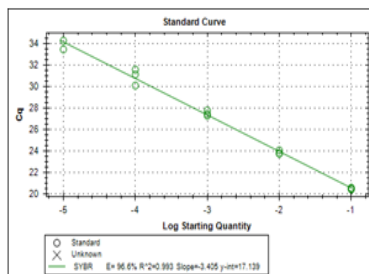*TIP41*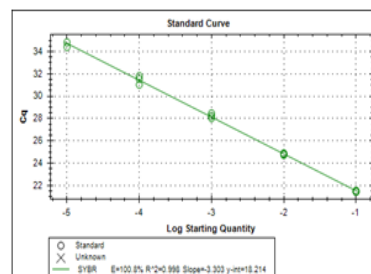*CAC*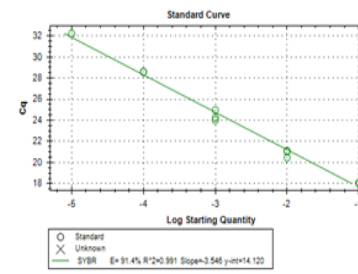*EXP*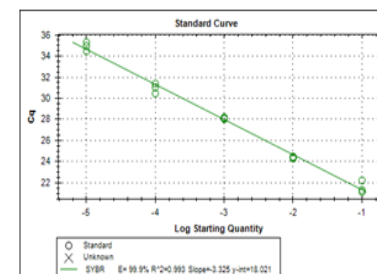*β TUB*

Supplement: S4 Fig — (PDF) [file pone.0284456.s004.pdf]

Additional Support Fig 5.

**A**

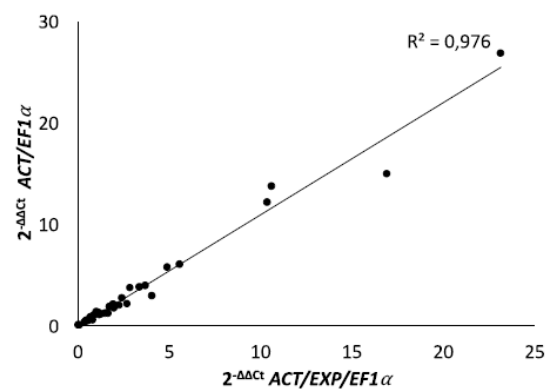

**B**

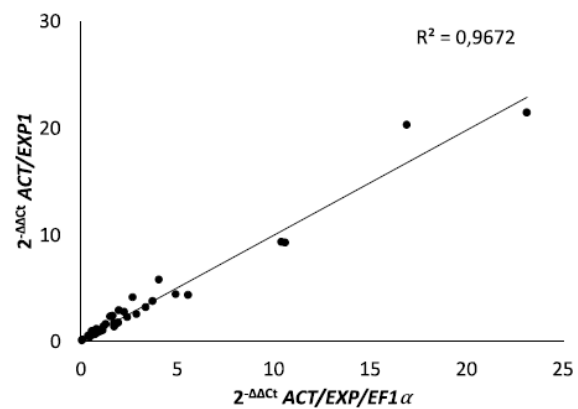

Supplement: S5 Fig — (PDF) [file pone.0284456.s005.pdf]
