# Supplementary material for: The selection and validation of reference genes for quantitative real-time PCR studies in near-isogenic susceptible and resistant tomato lines, infected with the geminivirus tomato curly stunt virus
Source: PLoS One. 2023 Jul 27;18(7):e0284456. doi: 10.1371/journal.pone.0284456 (PMC10374155; doi:10.1371/journal.pone.0284456)
Supplement: S3 Fig — (PDF) [file pone.0284456.s003.pdf]

Additional Supporting Figure 3

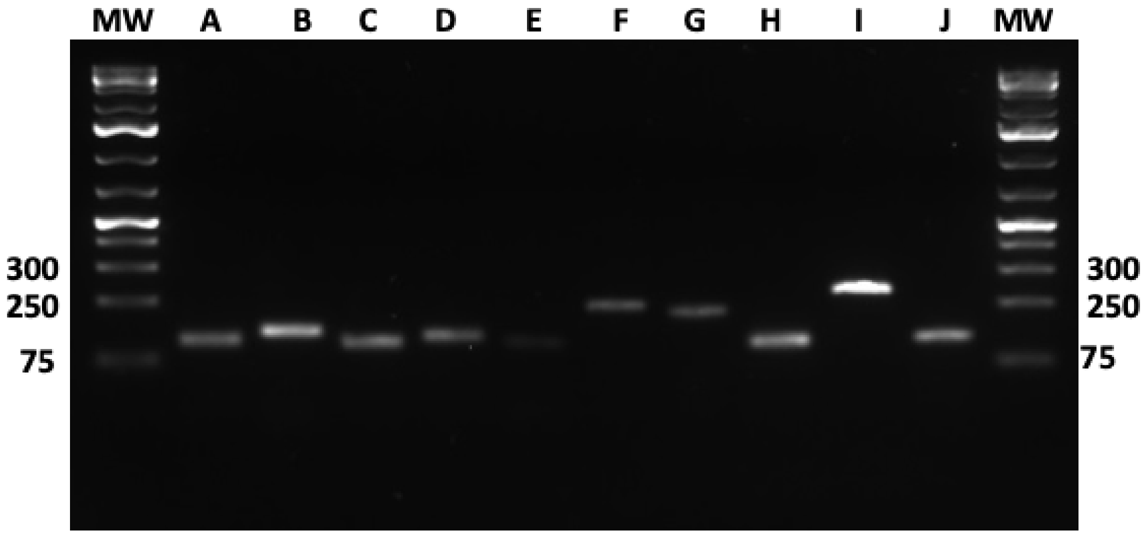

S2 Fig. (A) Amplification curves and (B) Melting curves generated after amplification of the candidate reference genes

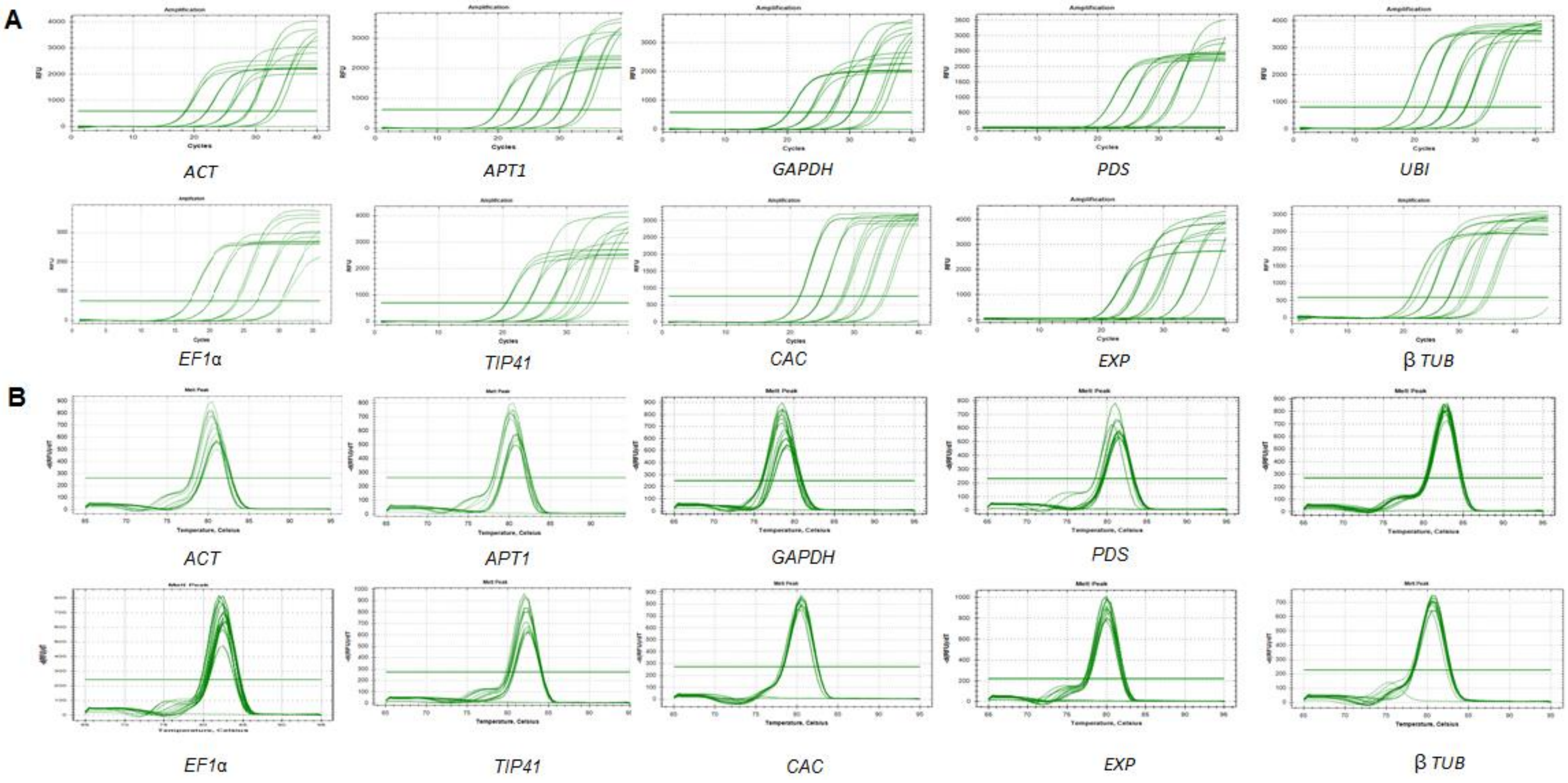

S3 Fig. Standard curves for ten candidate reference genes, generated from a 10-fold dilution series of cDNA.

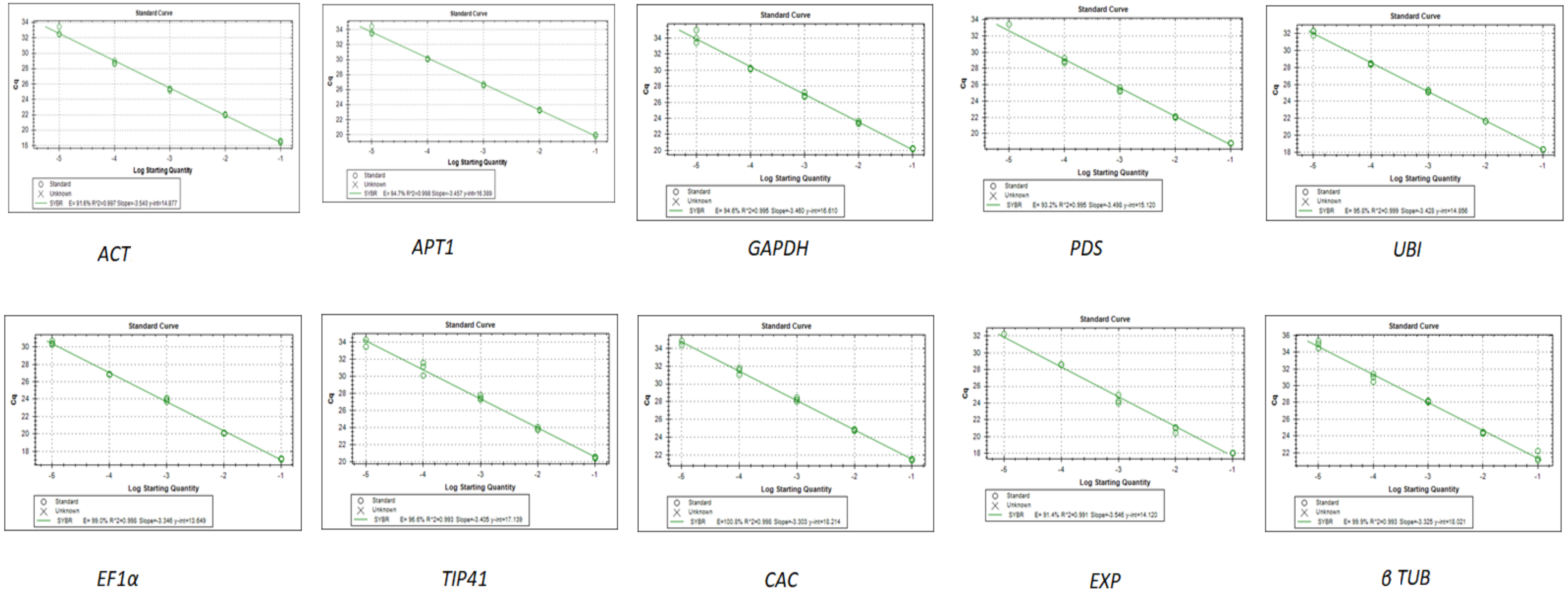

S4 Fig. Scatter plots of correlation

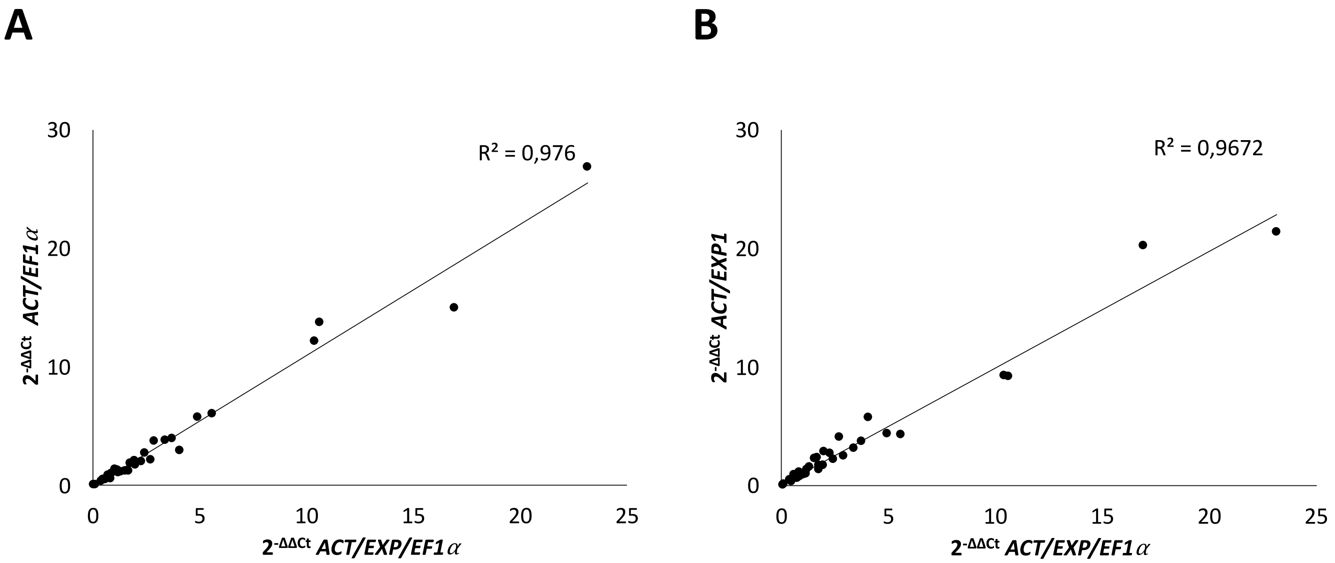

S4 Fig. Scatter plots of correlation between gene expression normalized against *ACT/EF1α* and *ACT/EXP* to gene expression normalized against *ACT/ EXP/EF1α* (n = 24, 12 infected and 12 mocks).
